# Migration control: a distance compensation strategy in ants

**DOI:** 10.1007/s00114-016-1386-8

**Published:** 2016-07-18

**Authors:** Thomas A. O’Shea-Wheller, Ana B. Sendova-Franks, Nigel R. Franks

**Affiliations:** 1 School of Biological Sciences, University of Bristol, Life Sciences Building, 24 Tyndall Avenue, Bristol, UK; 2 Department of Engineering Design and Mathematics, UWE Bristol, Frenchay Campus, Coldharbour Lane, Bristol, UK

**Keywords:** Cost-benefit trade-offs, Decentralised systems, Ecological robustness, Group migration, *Temnothorax albipennis*

## Abstract

Migratory behaviour forms an intrinsic part of the life histories of many organisms but is often a high-risk process. Consequently, varied strategies have evolved to negate such risks, but empirical data relating to their functioning are limited. In this study, we use the model system of the house-hunting ant *Temnothorax albipennis* to demonstrate a key strategy that can shorten migration exposure times in a group of social insects. Colonies of these ants frequently migrate to new nest sites, and due to the nature of their habitat, the distances over which they do so are variable, leading to fluctuating potential costs dependent on migration parameters. We show that colonies of this species facultatively alter the dynamics of a migration and so compensate for the distance over which a given migration occurs. Specifically, they achieve this by modulating the rate of ‘tandem running’, in which workers teach each other the route to a new nest site. Using this method, colonies are able to engage a larger number of individuals in the migration process when the distance to be traversed is greater, and furthermore, the system appears to be based on perceived encounter rate at the individual level. This form of decentralised control highlights the adaptive nature of a behaviour of ecological importance, and indicates that the key to its robustness lies in the use of simple rules. Additionally, our results suggest that such coordinated group reactions are central to achieving the high levels of ecological success seen in many eusocial organisms.

**Electronic supplementary material:**

The online version of this article (doi:10.1007/s00114-016-1386-8) contains supplementary material, which is available to authorized users.

## Introduction

Cost-benefit trade-offs are common to almost all life history strategies. Animals need to be able to respond to hostile environmental conditions such as extremes of temperature and predation risk (Creel & Winnie 2005; Hunter 2005; Nonacs & Dill 1990). Indeed, many life history events involve increased exposure to hazards, but are nevertheless crucial for survival, making a degree of risk essentially unavoidable (Thirgood et al. 2004; Cerdá et al. 1998; Franks & Fletcher 1983; Guillemette et al. 2012). As a consequence, strong selection pressures are likely to exist for strategies that mitigate the dangers of high-risk but necessary behaviours, and a myriad of such responses may be seen in nature. These range from vigilance behaviour in meerkats (le Roux et al. 2009) and camouflage in numerous species of mammal (Stevens & Merilaita 2009) to escape reflexes and group defence in migrating invertebrates (Wine & Krasne 1972; Scho 2005).

Social insects also face such challenges, but their responses often become manifest at the group or colony level, rather than at the individual level (Mlot et al. 2011). This enhanced cooperative ability is thought to be a central tenet of their remarkable ecological success (Hunt et al. 2010). A key aspect of the ability of social insect colonies to coordinate group behaviours is the need for reliable and rapid information relating to the immediate environment, with examples encompassing sensing of temperature (Rosengren et al. 1987), perception of the intensity of predation (O’Shea-Wheller et al. 2015; Whitford & Bryant 1979) and the detection of potentially hostile conspecifics (Franks & Fletcher 1983). Another factor that influences colony exposure to risk is the distance that must be travelled either as part of foraging, or during migration behaviours. Distance-cost scaling can be observed in vertebrate foraging (Lima 1985), and is likely also to apply to social insects, as the time that individuals spend away from the safety of their nest necessarily increases as a function of distance travelled (Traniello 1989). Consequently, many species of social insects have evolved methods of estimating distances to food sources and potential new nest sites. For example, there is evidence that bees measure distance using both optic flow odometry and path integration, helping them to find desirable resources and relay their location to other colony members (Srinivasan et al. 1997; Chittka et al. 1995). Within the *Formicidae*, the desert-dwelling ant *Cataglyphis fortis* also uses path integration, but based on a combination of celestial compass cues and pedometry, allowing reliable navigation in its expansive and relatively featureless environment (Wittlinger et al. 2006; Wehner 2009). However, there are fewer studies relating to how social insects may use such information to reduce mortality and risk of disorientation at the group level.

The ant *Temnothorax albipennis* is a model species that is well suited to testing how an increase in perceived risk may influence colony-level behaviour. Colonies of this species live within fragile rock cavities in a highly heterogeneous environment, and will migrate into better structures under both field and laboratory conditions, whenever the opportunity is presented. Colony migrations to new nest sites thus make up a crucial part of the species’ life history, but at the same time represent a high-risk process, whereby the vulnerable brood and all colony members are exposed (Dornhaus et al. 2004). Furthermore, the distance between the old and new nests influences total migration rate and thus is inseparably linked to the total level of risk during any given migration. Migration distances are highly variable; colonies will migrate into a better quality nest even if it is as far as 2.85 m from their current one, or as close as 10 mm (Franks et al. 2008). As such, it seems likely that *T. albipennis* colonies employ strategies to minimise risk when conducting migrations over longer distances, and there are several candidate behaviours that may be involved in this. Perhaps most important in ensuring the continuity and speed of migrations is a behaviour known as tandem running. This involves an ant that knows the location of the new nest leading another colony member to that nest and thus teaching it the route, and is implicated in migration rate and distance assessment (Hölldobler & Wilson 1990; Franks & Richardson 2006).

Here, using migrating colonies of *T. albipennis*, we aim to test whether the distance that must be travelled to a new nest affects the expression of important migration behaviours. Longer migration distances are likely to increase the time that workers spend travelling between nests, prolonging their exposure period, and thus the degree of risk (Langridge et al. 2008). Hence, we predict that colonies will be able to compensate for distance. To assess any such strategies, in our experiments, we provide colonies with both ‘near’ and ‘far’ new nest sites and allow them to migrate. During migrations, their current nests are left intact, acting to prolong the decision-making process and allowing us to measure more finely the dynamics of colony relocation (Dornhaus et al. 2004).

In this study, we focus on several key migration behaviours. First, we record the amount of forward and reverse tandem running conducted by colonies. Tandem running involves informed workers leading naïve colony mates to a new nest site (forward tandem running), or from the new site back to their old nest (reverse tandem running), in order to teach them the relative locations of each (Franks & Richardson 2006). Tandem runs are crucial to the progression of migrations, as movement requires an active ‘corps’ of spatially informed workers (Franks et al. 2009). Second, we quantify aspects of quorum sensing. This is a process in which ants assess the number of nest mates within a potential new nest site, and once the population in a candidate site reaches a certain value, termed the ‘quorum threshold’ ants will switch from slow tandem running to rapid social carrying, a transition known as ‘quorum attainment’ (Franks et al. 2015). Quorum sensing, by which ants assess the quorum threshold, is thus central to solidifying a colony’s target nest choice and in implementing the subsequent migration process. Third, we measure the per capita rate of social carrying, in which workers, informed of a new nests location via tandem running, rapidly carry their colony mates to the new nest site, once a quorum threshold has been achieved (Planqué et al. 2007). Social carrying allows migrations to proceed rapidly after a decision has been made, as it is three times faster than tandem running (Planqué et al. 2007).

We hypothesise that when nest sites are further away, colonies will increase the use of behaviours implicated in the rapidity of migrations, such as forward and reverse tandem running. Furthermore, it may be postulated that over longer distances, workers might employ a lower quorum threshold, in order to commit to a new nest more rapidly, begin carrying nest mates sooner, and thus expedite the migration. By analysis of such metrics, in concert with migration rates over different distances, we aim to assess how, and indeed whether, colonies make adjustments in order to counteract the increased danger posed by longer migrations.

## Materials and methods

### Colonies

We collected 30 colonies of *T. albipennis* from the Isle of Portland, Dorset (50.547889°N, −2.448251°W), on 22 January 2015. Each contained a queen, from 45 to 397 workers, and 11 to 356 brood items (eggs, larvae and pupae). No permission for collection was required as *T. albipennis* is not a protected species and colonies were taken from a disturbed quarry area with free public access. However, care was taken to keep disturbance of the population to a minimum by use of a rota based on the dates and locations of previous collections. Colonies were maintained under standard laboratory conditions, and all nests were housed in plastic Petri dishes with Fluon-coated sides to avoid worker escape. Ants were fed with *Drosophila melanogaster* weekly, and allowed to forage for water and honey solution *ad libitum* in accordance with established upkeep methods (Dornhaus et al. 2004).

Nests of two different qualities (Franks et al. 2003) were used in the experiments, both with an internal cavity area of 60 × 35 mm. ‘Poor’ quality nests, in which all colonies were initially housed, had 1-mm-high walls, a 6-mm-wide entrance and a clear cover. ‘Excellent’ quality nests, into which colonies were able to migrate during trials, had 2-mm-high walls, a 1-mm-wide entrance and a red filter cover. The poor nests had transparent lids to simulate cavities that were compromised by high light levels. The use of red filters in excellent nests ensured lower perceived light levels (Ogawa et al. 2015). This made them preferable to migrating ants, but at the same time did not obscure the observation of individuals within these nests (Franks et al. 2006).

### Treatment groups

After collection, all colonies were allowed to migrate into initial poor quality nests and left to acclimatise for a period of 7 days. This acclimatisation interval was used as colonies gain experience with multiple migrations, leading to changes in decision speed, and such experience is lost only after 7 days of inactivity (Langridge et al. 2004). Prior to initiation of the experiments, we grouped each of the 30 colonies into sets of 3 based on size, then randomly assigned each colony to one of the following three experimental groups: the ‘near nest’ group, the ‘distant nest’ group and the ‘delayed near nest’ group. Each colony underwent only the experimental trial to which it was assigned and emigrated only once. New nests in the ‘delayed near’ treatment were no further away than in the ‘near’ treatment but were only placed into arenas after a substantial time interval of 90 min. This 90-min interval was chosen as it allowed time for scouts to reach and explore the area in which the new nest would eventually be placed. The purpose of this treatment was to assess whether the time a colony spent scouting in an area before a new nest site was found affected the perceived accessibility of such a nest, and thus elicited changes in migration behaviour.

### Experiment protocol

**Fig. 1:**
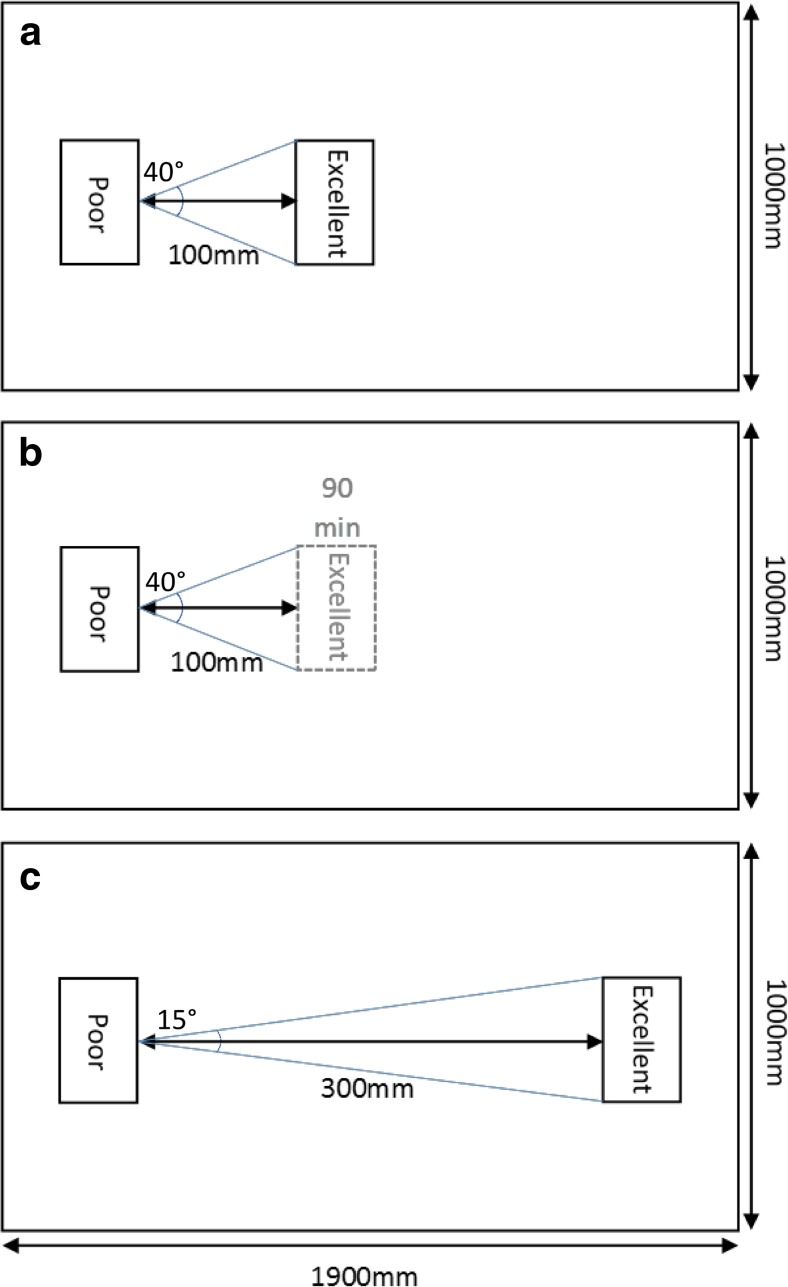
Layout of experimental arenas for **a** the near treatment; **b** the near delayed treatment, in which the excellent new nest was only placed into the arena after 90 min; and **c** the distant treatment. Starting nests housing *Temnothorax albipennis* ant colonies were of poor quality and were not destroyed, allowing voluntary emigrations to occur. The search angles leading to discovery of new nests are indicated; all new nest sites were of excellent quality. Figure aims to demonstrate nest quality setup and thus is not to scale

A total of 30 emigrations were conducted over a 19-day period from the 2nd to the 20th of February, 2015. Experiments were performed in 1900 × 1000-mm plastic arenas with Fluon-coated sides (Fig. 1). These large arenas were built in order to ensure an extensive potential scouting area, and thus better simulate natural conditions, as ants of this species have been known to migrate into new nest sites up to 2850 mm away (Franks et al. 2008). Two trials were conducted each day simultaneously, in order to limit the total experimental duration. This was desirable, as *T. albipennis* colonies show seasonality, and thus, shorter experimental periods are required for behavioural consistency (Franks et al. 2006). The order of treatments used was also randomised to make sure that no unintended time-related biases occurred. Prior to each trial, the arenas were cleaned with alcohol and water to remove any odours, and lit evenly using an overhead LED lighting unit (colour rendering index 85, luminous flux 4100).

Occupied poor nests were placed into fixed positions in the arenas, and new excellent nests were placed at set distances from these, dependent on the treatment group. For the near nest group, new nests were positioned so that their entrance was 100 mm from the starting nest, whilst for the distant nest group, new nests were set up at a distance of 300 mm (Fig. 1a, c). The potential search angles that allowed discovery of these nests were measured by extrapolating the front face size of new nests over the distance to the original nest entrance and then quantifying the angle with a protractor (Fig. 1). New nests were placed into the open arenas as the use of bridges or channels would artificially lead ants towards them, and thus remove any potential differences in nest discovery frequency. The delayed near nest treatment was also set up with a 100-mm separation to ensure consistency. In the delayed near treatment, the new nest was only placed into the arena 90 min after the nest occupied by the colony, and recording began only after the 90-min lag period (Fig. 1b). During the movement of nests into the arenas, any scouts that were outside their nests in the original Petri dishes were placed onto the tops of the starting nests within the arenas, using a fine paint brush to minimise disturbance. This was done in order to avoid any scouting discrepancies caused by different initial locations of scouts. From this point, we allowed emigrations to proceed whilst recording the number of workers and brood in each nest every 10 min for the first 180 min. We also recorded, via direct counts, the timings of new nest discovery, first tandem run, quorum attainment (measured by first instance of social carrying) and the amount of time that first discoverers spent in each new nest. Additionally, we quantified the per capita number of forward and reverse tandem runs, relative quorum threshold and per capita number of worker and brood social carries, with tally counts. Relative rather than absolute quorum threshold was used for consistency and comparability, as quorum threshold scales linearly with colony size (Dornhaus and Franks 2006). As migrations were generally slow and all behaviours were distinct, recordings were made manually, in accordance with established laboratory techniques (O’Shea-Wheller et al. 2015). If migrations were not completed within 180 min, colonies were left for a further 24 h before returning them to their holding dishes. For incomplete migrations, all relevant behaviours that occurred up to the end of the 180-min observation period were included in the analyses, but no data were recorded after this point.

### Data analysis

In the analysis of all factors, Shapiro-Wilk tests were employed to determine if the data differed significantly from a normal distribution. For normally distributed data with homogeneous variance, we used one-way ANOVA with Tukey’s HSD post hoc tests to analyse differences between treatment groups. Where the data was found to be not normally distributed, we transformed it using either arcsine square root, or log10 transformations, dependent on its skewness. This was done to ensure homogeneity of statistical testing methods. Means quoted from this data were reverse transformed after calculation. Transformations did not alter the pattern of statistical significance, confirmed by testing data before and after transformation, with both parametric and non-parametric tests. For per capita forward and reverse tandem running, relative quorum threshold and the per capita rate of social carrying, data were normalised for colony size. This was achieved by calculating values in proportion to colony population, in order to determine the per capita expression of a behaviour based on the number of available individuals.

We used a Kaplan-Meier survival analysis to assess the effect of treatment group on the mean percentage of ants and brood building up in new nests over time. We used means of the data to produce a cumulative survival function, and ants and brood that moved after 180 mins were treated as censored points. Pairwise comparisons were made using the Mantel-Cox test, for which alpha was 0.016 based on a Bonferroni’s correction for the three possible pairwise comparisons between treatment groups. All analyses were conducted in SPSS (Release version 21.0.0.0, IBM Corporation and other(s) 1989, 2012).

## Results

### Behavioural phases and tandem running

The timings of nest discovery (one-way ANOVA, *F*_2,27_ = 17.439, *P* < 0.001), first tandem run (one-way ANOVA, *F*_2,27_ = 13.384, *P* < 0.001) and quorum attainment (one-way ANOVA, *F*_2,27_ = 7.543, *P* = 0.023) were significantly affected by treatment group. This was due to the timings of nest discovery (means near = 11.94 min, near delayed = 6.66 min, distant = 14.15 min), first tandem run (means near = 32.80 min, near delayed = 19.74 min, distant = 57.82 min) and quorum attainment (means near = 69.26 min, near delayed = 54.12 min, distant = 115.57 min) within the near nest and delayed near nest treatments occurring significantly earlier than in the distant nest treatment. Principally, these results can be explained by the increased difficulty of finding new nests in the distant treatment and consequent slowing of migration progression.

**Fig. 2:**
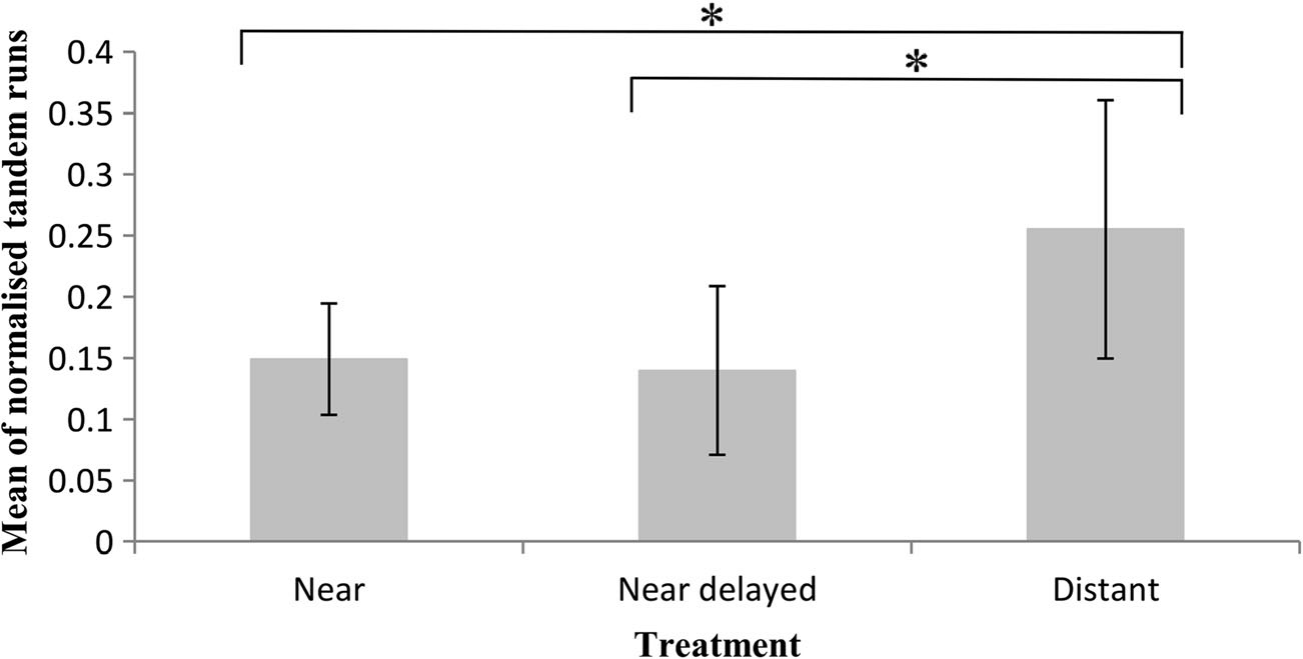
Mean number of tandem runs (per capita) across treatments. Significant differences between groups (*P* < 0.05), based on ANOVA analysis and Tukey’s HSD post hoc tests, are indicated with *asterisks. N* = 10 colonies were used in each treatment; *error bars* indicate 95 % confidence intervals

The per capita number of tandem runs also varied significantly between groups (one-way ANOVA, *F*_2,27_ = 4.559, *P* = 0.020). Specifically, there was a higher per capita number of tandem runs in the distant nest treatment than in either the near nest (Tukey’s HSD, mean difference = −0.106, *P* = 0.048) or delayed near nest (Tukey’s HSD, mean difference = −0.115, *P* = 0.030) treatments (Fig. 2). This was likely caused by a lower rate of independent nest discovery by workers in the distant treatment; thus, more tandem runs were needed to reach a quorum threshold in this group.

The relative quorum threshold (one-way ANOVA, *F*_2,27_ = 1.240, *P* = 0.305), per capita rate of social carrying (one-way ANOVA, *F*_2,27_ = 0.570, *P* = 0.572) and time that first discoverer spent in nest (one-way ANOVA, *F*_2,27_ = 1.179, *P* = 0.323) did not differ significantly between treatments. Such a consistent relative quorum threshold is contrary to our hypothesis that lower thresholds may be employed over longer distances, and may be accounted for by the move-to-improve conditions used in our experiments (Dornhaus et al. 2004). Furthermore, the lack of change in social carrying rates may possibly be explained by the comparable relative quorum thresholds between treatments. Similar quorums suggest that the average numbers of workers in the new nests at the beginning of carrying were similar in each treatment, and these workers were then the ones that contributed to the carrying process. As such, numbers of carriers were unlikely to differ between treatments, despite changes in tandem running. Lastly, as new nest qualities were always excellent, it is not necessarily surprising that discoverers spent a consistent time within them, regardless of distance. The percentage of ants in the new nest at the time of first tandem run (initial independent discoveries; one-way ANOVA, *F*_2,27_ = 1.441, *P* = 0.254; means near = 2.750 %, near delayed = 2.165 %, distant = 1.184 %) and the number of reverse tandem runs per capita (one-way ANOVA, *F*_2,27_ = 0.355, *P* = 0.704) also did not differ significantly between treatments.

### Progression of migrations

**Fig. 3:**
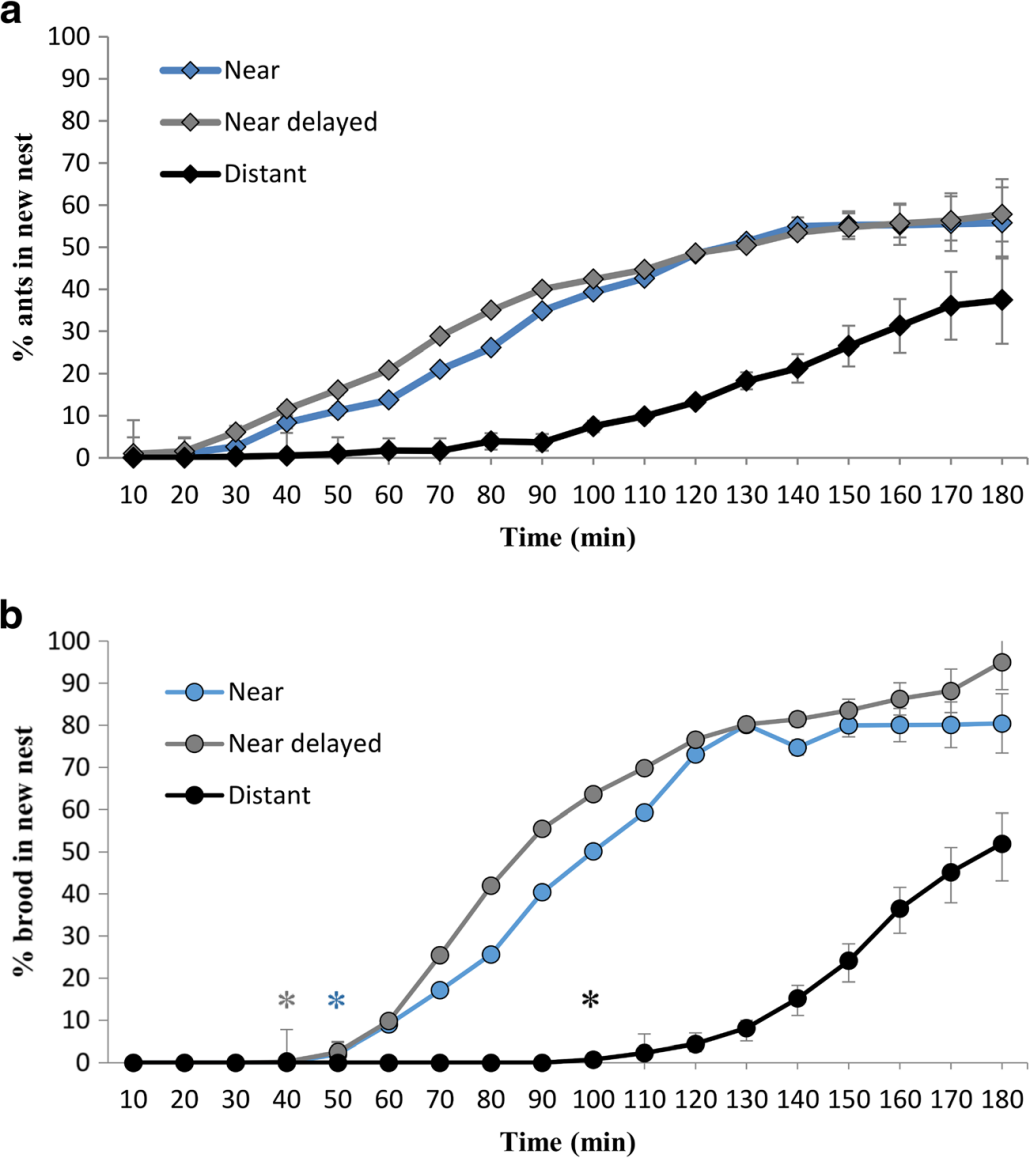
Mean percentage of total colony workers (**a**) and brood (**b**) in the new nests over time for each of the three treatment groups. *Blue lines* indicate the near treatment, *grey lines* indicate the near delayed treatment and *black lines* indicate the distant treatment. In the near delayed treatment, colonies were allowed to scout for 90 min but with no new nest present until time 0. The mean represents *n* = 10 colonies for each time point; *asterisks* indicate the mean time of first brood transport (quorum attainment), in each correspondingly coloured group. All *error bars* indicate 95 % confidence intervals

The average percentage of ants building up over time in the new nests was significantly different between groups (Mantel-Cox test statistic = 17.349, *df* = 2, *P* < 0.001). Specifically, this was explained by differences between the near nest and distant nest treatments (Mantel-Cox test statistic = 13.708, *df* = 1, *P* < 0.001) and delayed near nest and distant nest treatments (Mantel-Cox test statistic = 15.700, *df* = 1, *P* < 0.001). However, the near nest and delayed near nest treatments were not significantly different (Mantel-Cox test statistic = 0.375, *df* = 1, *P* = 0.540; Fig. 3). This suggests that the longer journey to nests in the distant treatment lead to a reduction in migration rate, but that this was not the case in either the near or delayed near treatments, both being equidistant from the original nest.

The average percentage of brood building up in the new nest over time was also significantly different between groups (Mantel-Cox test statistic = 69.132, *df* = 2, *P* < 0.001). Significant differences occurred between the near nest and distant nest treatments (Mantel-Cox test statistic = 46.416, *df* = 1, *P* < 0.001) and delayed near nest and distant nest treatments (Mantel-Cox test statistic = 72.298, *df* = 1, *P* < 0.001). However, again the near nest and delayed near nest treatments were not significantly different (Mantel-Cox test statistic = 3.375, *df* = 1, *P* = 0.066; Fig. 3). These results point to the same trend as seen in worker movement, whereby only increased distance led to a slower migration. Notably, overall brood transport was faster than the movement of workers. This is because brood items are immobile, and due to their vulnerability, are rapidly moved to the new nest with preference over adults (Langridge et al. 2008).

**Fig. 4:**
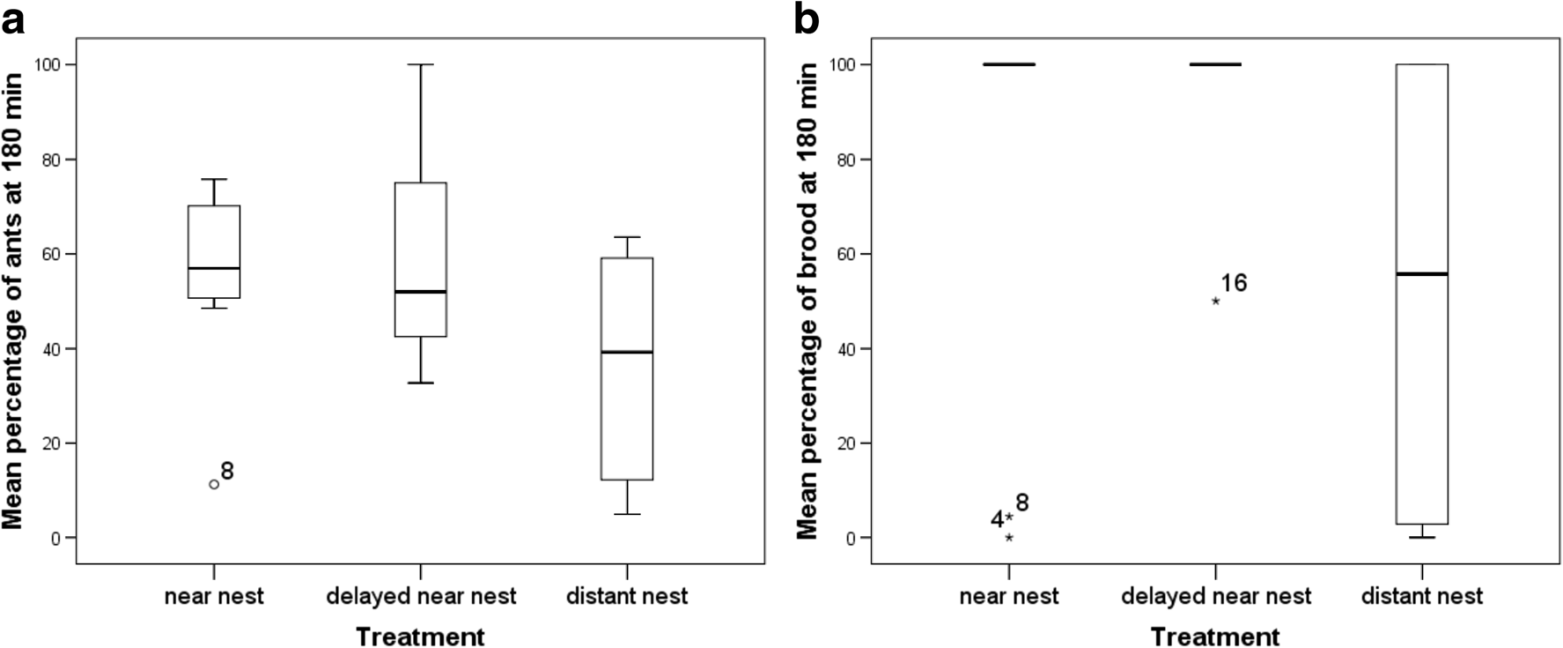
Boxplots for percentages of ants (**a**) and brood (**b**) in the new nests at 180 min, *n* = 10 colonies per treatment. *Boxes* are cut at the median, with the upper and lower limits of the box representing the upper and lower quartiles, respectively. Outliers (further than 1.5 times the interquartile range from the median) are marked with *circles* (ants) and *asterisks* (brood); *numbers* indicate colony IDs

The percentages of workers (one-way ANOVA, *F*_2,27_ = 2.798, *P* = 0.079) and brood (one-way ANOVA, *F*_2,27_ = 3.178, *P* = 0.058) in the new nests at the final time points did not differ significantly between treatment groups, but it must be noted that values in the distant treatment were still lower for both measures, and both *p* values were small even if above 0.05 (Fig. 4). This is indicative of a partial, but incomplete, adjustment of migration rates by the end of the process.

## Discussion

We show that colonies of *T .albipennis* can respond to differing migration conditions via the regulation of tandem running behaviour. Specifically, we found that colonies will increase the per capita number of tandem runs used during an emigration when the distance to be traversed is greater (Fig. 2). In this way, migration transit time is minimised and exposure period is reduced. Furthermore, we found that the end result of migrations after 180 min across colonies was comparable regardless of the distance travelled (Fig. 4), but that the underlying dynamics varied significantly (Figs. 2 and 3). This indicates that colonies increase the rate of tandem running when a new nest is further away, in order to expedite the migration process, by increasing the rate at which workers discover the new nest (Stuttard et al. 2015). Our results demonstrate that travel distance significantly affects colony migration strategy in move-to-improve scenarios, and additionally, they highlight the importance of perception at the individual level in influencing collective behaviour.

Independent discoveries—that is ants finding the new nest without being led to it—were less frequent in the far nest treatment than in the near or near delayed treatments. This was characterised by the buildup of ants in the new nests prior to the initiation of tandem running, as all individuals present at this time made independent discoveries. The numbers of ants in new nests at the time of the first tandem run did not differ significantly between treatments, suggesting that tandem running began at a set nest population. However, crucially, along with quorum attainment, the first tandem run occurred later in the distant nest treatment (Fig. 3). This showed that more time was required to reach such a population and was indicative of a lower discovery rate when the nest was further away. Furthermore, the search angle leading to discovery of the front surfaces of the new nests in the near and delayed near treatments (40°), was more than double that of the distant treatment (15°; Fig. 1), indicating that distant nests were harder to find, and additionally presented smaller vertical landmarks. Indeed, such differences are ecologically relevant to the probability of nest discovery, principally because ants of this species do use visual landmarks, and upon contact with the front of a nest, scouting workers will often employ thigmotaxis to locate its entrance (Basari et al. 2014; Hunt et al. 2014).

As distant nests were more difficult to discover, the role of increased numbers of tandem runs was clear; they led to an increase in the discovery rate and thus generated a larger pool of informed workers, potentially able to contribute to the carrying stage of the migration (Franks & Richardson 2006; Planqué et al. 2007). Conversely, the absence of this process in the delayed near nest treatment can be explained because such nests were no further away than in the near treatment. Without an increased travel distance, the independent discovery rate and time taken to recruit naive ants to the new nest would not have differed between workers in the two near treatments. Hence, there was little stimulus for colonies to compensate based on perception at the individual level, despite delayed nest discovery. This is advantageous, as in their natural habitat, nest discovery times are likely to be more variable due to non-uniform environmental features. In light of this, it is reasonable to assume that colonies accounted for the increased distance between nests without the use of discovery time as a proxy.

Although the use of landmarks and pedometry have been implicated in nest location during tandem running (Franklin & Franks 2012; Basari et al. 2014), here, a system based on independent discovery rate provides a more parsimonious explanation for our results (Pratt 2004). When nests are nearby, exploring workers are likely to find them relatively easily. However, this is not the case when they are distant. Hence, more tandem runs are required over longer migration distances simply because independent discoveries are rarer, and thus, more ants must be led to a nest before a quorum threshold can be reached. In this way, workers do not measure distance directly, but instead respond to quorum attainment, a factor that in this case was influenced by distance (Pratt 2004). It is known that *T. albipennis* workers use encounter frequency to measure nest size and quorum threshold (Stuttard et al. 2015; Pratt 2004) and that tandem running will continue up until the point that a quorum threshold is reached (Planqué et al. 2007). Lower discovery rates extend the pre-quorum period, as seen in the distant treatment, and so a greater proportion of ants in the new nest are led there by tandem runs. As such, the greater number of tandem runs is likely a self-regulating process, controlled by quorum attainment alone, rather than by actual distance measurement.

The aforementioned self-organisation hypothesis is consistent with earlier work on *T.albipennis*, *T.curvispinosus* and *T.rugatulus*, suggesting that when independent nest discoveries were more common, the rate of tandem running decreased (Mallon et al. 2001; Pratt 2008). Notably, whilst these studies used forced migrations, involving the destruction of colonies’ original nests, our own data appear to corroborate their findings in relation to environmentally common move-to-improve migrations, in which tandem running plays a greater role (Dornhaus et al. 2004). Tandem runs to near nests are rare or absent under emergency migration conditions (Dornhaus et al. 2004), but in our experiments, all colonies in the near treatment still engaged in this behaviour. Indeed, there is a clear advantage to doing so under benign conditions, as during the tandem running stage, colonies are still open to switching to a better nest site if one is discovered, whilst this is no longer the case once they commit to carrying (Franks et al. 2007; Sasaki et al. 2015).

There is a substantial body of literature implying that for any given migration, quicker is better (Langridge et al. 2008; Scharf et al. 2012; Franks et al. 2009; Franks et al. 2008), and colonies in our experiment did attempt to expedite migrations. In spite of this, we found that longer migration distances still led to slower movement, even with the partial rescuing of migration rate by increased tandem running (Figs. 3 and 4). This highlights the often-incomplete nature of compensatory behaviours, and suggests that for *T.albipennis* colonies, increased migration distance may always impart some degree of cost.

Whilst tandem running is a crucial behaviour implicated in regulating migrations (Franks & Richardson 2006), there is also evidence to suggest that reverse tandem runs; from the new to the old nest, play a role in modulating migration rate (Franks et al. 2009). However, we found no significant effect of migration distance on the number of reverse tandem runs. This result may be explained by the findings of previous empirical studies, showing that increased numbers of reverse tandem runs are induced only by disorientation of active scouts. Such studies indicate that the primary purpose of reverse tandem running is to re-engage ‘lost scouts’ in the migration process, rather than to train naïve workers (Planqué et al. 2007; Franks et al. 2009). In our experiments, distance was the key limiting factor; thus, it was preferable to engage as many new workers in the tasks of recruitment and carrying, a process achieved by the use of forward tandem runs alone (Franks & Richardson 2006). As such, it is probable that colonies facultatively alter both forward and reverse tandem running to control migration rate, dependent on the particular challenges that threaten to curtail it.

Building on the work of Pratt (2004), we show the potential for colonies to modulate their behaviour in response to distance, using a decentralised system based on perception at the individual level. Indeed, it may well be argued that for the purposes of migration in *T.albipennis*, such a comparatively simple system provides superiority in both rapidity and robustness when juxtaposed against more sophisticated distance assessment methods (Thiélin-Bescond & Beugnon 2005). Furthermore, the specific costs addressed by this process are of particular ecological significance, as migration distances vary widely in nature (Franks et al. 2008).

Our work demonstrates an example of colony-level risk mitigation behaviour, which, taken with other such examples (O’Shea-Wheller et al. 2015; Gordon et al. 2007), further illustrates the enhanced ability of social insects to adapt and respond to risks over a range of spatial and temporal scales. Moreover, as is the case with numerous other complex systems (Siljak 2012; Zimmerman et al. 2014), the key to this behaviour lies in its decentralised control. Thus, we provide further evidence that the robustness of self-organised systems is one of the central factors enabling eusocial animals to meet ecological challenges.

## Electronic supplementary material

Below is the link to the electronic supplementary material.

Supplementary material 1 Raw data from the experiment (XLSX 180 kb)
